# Linkage Disequilibrium between Polymorphisms of ABCB1 and ABCC2 to Predict the Treatment Outcome of Malaysians with Complex Partial Seizures on Treatment with Carbamazepine Mono-Therapy at the Kuala Lumpur Hospital

**DOI:** 10.1371/journal.pone.0064827

**Published:** 2013-05-23

**Authors:** Soobitha Subenthiran, Noor Rain Abdullah, Joyce Pauline Joseph, Prem Kumar Muniandy, Boon Teck Mok, Chee Cheong Kee, Zakiah Ismail, Zahurin Mohamed

**Affiliations:** 1 Bioassay Unit, Herbal Medicine Research Center, Institute for Medical Research, Kuala Lumpur, Malaysia; 2 Department of Neurology, Kuala Lumpur General Hospital, Kuala Lumpur, Malaysia; 3 Medical Research Center, Institute for Medical Research, Kuala Lumpur, Malaysia; 4 Department of Pharmacology, Faculty of Medicine, University of Malaya, Kuala Lumpur, Malaysia; Kaohsiung Chang Gung Memorial Hospital, Taiwan

## Abstract

**Purpose:**

Carbamazepine (CBZ) is used as the first line of treatment of Complex Partial Seizures (CPS) in the Epilepsy Clinic, Neurology Department of Kuala Lumpur Hospital (KLH). More than 30% of the patients remain drug resistant to CBZ mono-therapy. CBZ is transported by the P-glycoprotein (P-gp). The P-gp encoded by the ABCB1 and ABCC2 genes are expressed in drug resistant patients with epilepsy. A few studies have shown significant association between CBZ resistant epilepsy and Linkage Disequilibrium (LD) with adjacent polymorphisms of these genes. Our study is aimed at determining the correlation between patients' response to CBZ mono-therapy to Single Nucleotide Polymorphisms G2677T and C3435T of the ABCB1 gene as well as G1249A and −24C>T of the ABCC2 gene.

**Method:**

314 patients with CPS were recruited from the Neurology Department of the KLH based on stringent inclusion and exclusion criteria, of whom 152 were responders and the other 162 were non-responders. DNA was extracted from their blood samples and Taqman technology for allelic discrimination was performed. Results were described as genotype frequencies. The SHEsis analysis platform was used to calculate linkage disequilibrium index and infer haplotype frequencies. Haploview was used to do permutation test to obtain a corrected p-value.

**Results:**

Resistance to treatment with CBZ mono-therapy was significantly associated with the 2677TT and the 3435TT genotypes while it was not significantly associated with the G1249A and −24C>T polymorphisms. The GCGC haplotype combination of the 2677G>T, 3435C>T, 1249G>A and −24C>T respectively was found to be extremely significant (p = 1.10e−20) with good drug response to CBZ mono-therapy.

**Conclusion:**

Linkage disequilibrium between the 2677G>T, 3435C>T, 1249G>A and −24C>T SNPs may be used as a reliable screening marker to determine the treatment outcome of CBZ mono-therapy with CPS irrespective of race or gender.

## Introduction

Epilepsy is a complex of seizures which arise from abnormal, excessive, synchronous and sustained discharge of a group of neurons. This in turn leads to persistent increase of neuronal excitability. 50% of all epilepsies are idiopathic while other causes of epilepsy are trauma, oxygen deprivation, tumors, infection, metabolic derangement, disorders of neuronal migration and monogenic epilepsies [1]. Complex Partial Seizures (CPS) start focally within the brain and causes impairment of consciousness [2]. It is the most common manifestation of Temporal Lobe Epilepsy (TLE) [3]. The incidence of epilepsy in Malaysia is 4–8 per 1000 population [4], while the percentage of CPS of idiopathic etiology is 5.7% [5].

Carbamazepine (CBZ) is used in the treatment of Generalized Tonic Clonic (Grand Mal), Partial and Generalized Secondary Seizures. CBZ is a derivative of Iminostilbene which is related to the tricyclic antidepressant family. This makes it suitable in the treatment of Bipolar Affective Disorder and Trigeminal Neuralgia [6].

In KLH, CBZ is used as the first line of treatment of CPS as it is known to control seizures effectively in most individuals and also because it is economical. CBZ is transported by binding to Albumin and α1-acid Glycoprotein (AGP), an acute phase protein. In normal individuals 75–85% of the drug is bound to these plasma proteins while 20–25% of it remains in its free form [7]. CBZ exhibits significant dose response variability so, individualizing dosage and determining if a person is resistant to the drug is essential. Drug resistance is known to be influenced by failure of drug transport which is thought to be governed by genetic factors. Three genes known to be involved in the drug transport are ABCB1, ABCC2 and RALBP1.

Conflicting results have been reported regarding the contribution of ABCB1 polymorphisms to CBZ disposition. The most common single nucleotide polymorphisms (SNPs) identified on the ABCB1 gene were C3435T, G2677T and C1236T. A study conducted on the Japanese population with epilepsy showed that the TT genotype of the C3435T and that the TTT haplotype of the 3 SNPs were associated with CBZ-resistant epilepsy [8]. However a meta-analysis done between drug resistant epilepsy in general showed no association to the C3435T SNP [9]. The same lack of association was also demonstrated in the Turkish population [10]. The correlation between the presence of “T” allele of the G2677T SNP and resistance to antiepileptic drugs (AEDs) were studied in a few countries such as Mosyagin I et al [11] in Germany, Grover S et al [12] and Vahab SA et al [13] in India and was previously thought to be non-significant. However, we did not include the C1236T SNP in this study as it has never shown any association with drug resistant epilepsy in any of the previous studies unlike the other SNPs.

The ABCC2 also known as the Multidrug resistance protein 2 (MRP2) is involved in the transport of antiepileptic drugs and was found to be up-regulated in the brain tissues of epileptic patients. It was hypothesized that genetic variations in the MRP2 gene would probably affect individual drug responses to CBZ. A study conducted in Korea showed that the “A” allele of the MRP2 single nucleotide polymorphism 1249G>A was associated with adverse neurological drug reactions to CBZ [14]. Another study which was conducted in Germany on 103 responders and 113 non-responders to first line antiepileptic drug therapy showed that the non-responders tended to carry the “T” allele of the −24C>T variant of the ABCC2 gene.

Malaysia is a multiracial country. Its peninsular comprises of three major ethnic groups namely, the Malays, Chinese and Indians who have lived in Malaysia for over five generations. They have been exposed to the same environmental factors such as diet and lifestyle. Recent association studies have shown no significant difference between the three ethnic groups in terms of drug response and disease susceptibility. The difference in findings of association studies among these SNPs which were conducted in other countries may be due to racial differences but after careful observation, it seems more likely due to the method in which the subjects were selected.

## Methods and Methodology

This study was approved by the Research Review Committee, Institute for Medical Research and Medical Research Ethics Committee (MREC), Ministry of Health Malaysia. The research was conducted in accordance to the principles expressed in the Declaration of Helsinki.

### 2.1 Recruitment of patients for study

Malaysian patients of Malay, Chinese and Indian were recruited from the Epilepsy Clinic, Neurology Department of KLH, weekly. There were a total of 40 to 50 patients who are followed up every Monday as KLH serves as a tertiary referral hospital. The patient files were identified prior to the epilepsy clinic and written informed consent was obtained from suitable study volunteers. Study volunteers who fulfilled the inclusion criteria were enrolled into the study. They were provided with clear verbal as well as written information about the study. Those enrolled into the study gave their informed consent voluntarily.

A total of 314 patients were recruited and enrolled into the study. One hundred and fifty six (49.7%), 84 (26.8%) and 70 (23.5%) were Malays, Chinese and Indians respectively. The study volunteers were diagnosed with CPS and secondary causes such as trauma, metabolic disorders, tumors and infectious causes were ruled out. As per the study design, 152 were responders to CBZ, while 162 were non-responders to CBZ. The study volunteers were required to answer a few questions regarding their family background (family history of epilepsy) their disease history (onset of the disease, characteristics as well of evolution of the seizure, frequency of attacks and treatment history) and progression (change in frequency of seizures after the onset of treatment) before they were assigned to the respective groups.

The responders were found to have normal MRI findings and mostly normal scalp EEG except a few patients who showed left or right temporal spikes. They were men and women between the age group of 18 to 60 years with no other co-morbid. They were already on an optimum dose of CBZ as a single drug therapy and were compliant to treatment. As they were being followed up every 3 months, we had chosen patients with no episodes of seizures in more than a year. We made sure that their serum CBZ levels were within the therapeutic range (17–51 µmol/L) to ensure compliance to treatment.

The non-responders were patients with CPS with similar MRI and EEG findings as the responders. They were men and women between the age group of 18 to 60 years with no other co-morbid. They were on a poly-therapy which included CBZ, at the time of recruitment. They were drug resistant patients. Their compliance to treatment while they were on CBZ mono-therapy was supported by their serum CBZ level before other antiepileptic agents such as Lamotrigine and Sodium Valproate were introduced.

Patients with other types of seizures were excluded from the study. Patients who were never treated with CBZ as well as those who had abnormal renal and liver function tests were also excluded from the study. In addition to this, patients who were on drugs which interacted with CBZ such as Alcohol, Macrolides, Anti Tubercular Drugs, Antifungal agents, Benzodiazepines, Anti Psychotics, Antidepressants, Calcium Channel Blockers, Cisplatin, H2 blockers, Cyclosporine, Doxorubicin, Tetracycline, Hormone Contraceptives, Anti Retro-viral drugs and Theophylline, were excluded from the study, to rule out confounding factors which may have influenced the outcome of treatment.

### 2.2 DNA extraction from whole blood

DNA extraction was done from the buffy coat using the Qiagene DNA extraction blood kit. DNA concentration and purity was quantified using the Nanodrop 2000 Spectrophotometer by Thermo Scientific.

### 2.3 Real Time PCR

Real Time PCR was run using SNP Specific Predesigned Taqman Probes as shown in Table 1 as well as Vic and Fam reporter dyes. The probe sequences and the assay codes are as shown in Table 2. DNA samples were diluted to a concentration of 10 nanogram/ µL. The assays were run using a reaction volume of 10 µL consisting of 5 µL of Applied Biosystems TaqMan GTXpress Master Mix, 0.5 µL of Applied Biosystems TaqMan Drug Metabolism SNP Genotyping assay, 1 µL of diluted DNA and 3.5 µL of Qiagene DNAse/RNAse free water. The reactions were run for 40 cycles at cycling temperatures as shown in Table 1, for 40 minutes.

**Table 1:** Step One Plus Real Time PCR setting.

Stage	Step	Temp	Time
Holding	DNA Polymerase Activation	95°C	20 sec
Cycling (40 cycles)	Denature	95°C	3 sec
	Anneal/Extend	60°C	30 sec

**Table 2:** Single Nucleotide Polymorphism (SNP) Probe sequence and Assay ID.

SNP	RS number	Probe Sequence	Assay code
G2677T	rs2032582	TATTTAGTTTGACTCACCTTCCCAG[C/T]ACCTTCTAGTTCTTTCTTATCTTTC	C_11711720D_40
C3435T	rs1045642	TGTTGGCCTCCTTTGCTGCCCTCAC[A/G]ATCTCTTCCTGTGACACCACCCGGC	C_7586657_20
c1249G>A	rs2273697	CAACTTGGCCAGGAAGGAGTACACC[A/G]TTGGAGAAACAGTGAACCTGATGTC	C__22272980_20
−24C>T	rs717620	ACAATCATATTAATAGAAGAGTCTT[C/T]GTTCCAGACGCAGTCCAGGAATCAT	C___2814642_10

### 2.4 Statistical Analysis

The statistical evaluation of the genotype data was performed with the Pearson's χ^2^ test using Statistical Product and Service Solutions SPSS 18.0 software (SPSS Inc., Chicago, Illinois, USA) to compare the genotypes frequencies between responders and non-responders to CBZ monotherapy. Fisher's exact test was used if the expected cell frequencies were lower than 5. Statistical significance was defined as p<0.05. Genotype frequencies at each locus were tested for Hardy-Weinberg equilibrium. On the basis of the observed frequencies of three SNPs, we used the SHEsis analysis platform to calculate linkage disequilibrium index and infer haplotype frequencies [15]–[17]. Permutation test was applied 1000 times to obtain a corrected p - value as shown in Table 3, using the Haploview software.

**Table 3:** Single Nucleotide Polymorphism (SNP) genotype distribution and response to Carbamazepine monotherapy.

	Genotype frequency (%)		*Permuted	*Permuted
SNP / Genotype	Responder	Non-responder	χ^2^	p
**ABCB1/ C3435T**				
C/C	35(0.22)	51(0.34)	12.06	0.007
T/T	68(0.42)	36(0.24)		
C/T	59(0.36)	65(0.43)		
				
**ABCB1/ G2677T**				
G/G	59(0.36)	144(0.75)	61.70	<0.001
T/T	57(0.35)	25(0.16)		
G/T	46(0.28)	13(0.09)		
				
**ABCC2/ G1249A**				
G/G	120(0.74)	124(0.82)	2.23	0.511
A/A	0(0.00)	1(0.01)		
G/A	42(0.26)	27(0.18)		
				
**ABCC2/ −24C>T**				
C/C	110(0.68)	102(0.67)	0.14	0.986
T/T	4(0.03)	6(0.04)		
C/T	48(0.30)	44(0.29)		

*1000 permutation applied.

### 2.5 Ethical Publication

We confirm that we have read the journal's position on issues involved in ethical publication and affirm that this report is consistent with those guidelines.

## Results

Among the 314 patients recruited, 156 were males while 158 were females with a mean age of 37.5±12.3 and 38.0±10.3 years among responders and non responders respectively. The responders were on a mean dose of 9.75±5.1 mg/kg/day and a mean serum CBZ level of 27.5±10.7 µmol/L.

**Figure 1:**
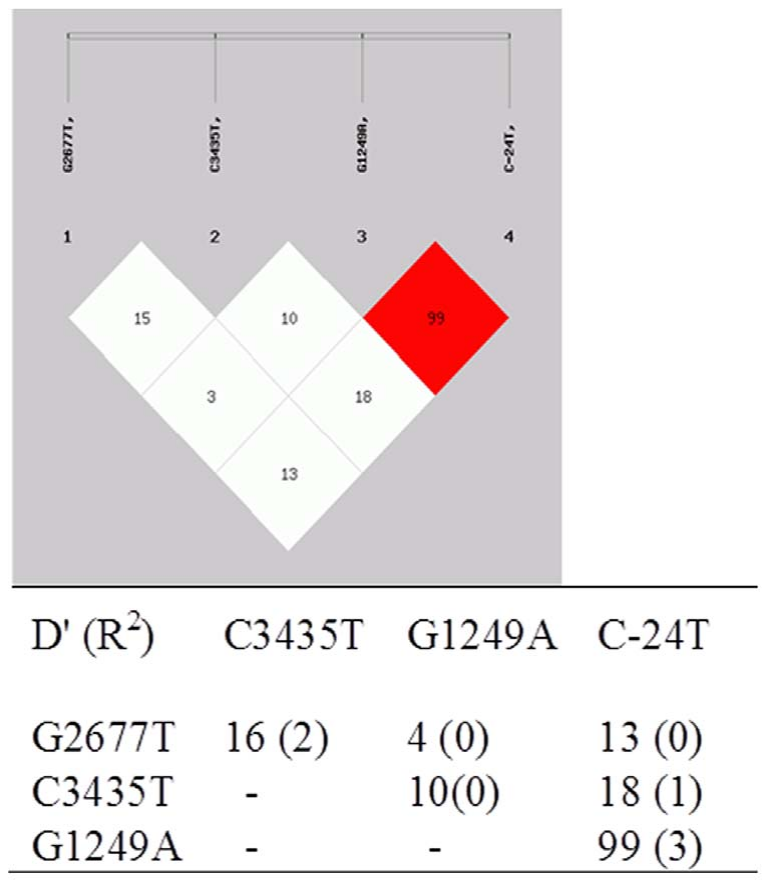
Linkage Desequilibrium test.

The distribution of all genotypes (wild-type, heterozygous and homozygous mutant variants) and genotype frequencies were as shown in Table 3. Genotype frequencies were in accordance to Hardy-Weinberg equilibrium model. Linkage Disequilibrium (LD) between haplotypes of the 2677G>T (ABCB1), 3435C>T (ABCB1), 1249G>A (ABCC2), −24C>T (ABCC2) were determined using the SHEsis program platform, as shown in Table 4. The C1236T SNP was not included in this study as it failed to show any association with drug resistant epilepsy in any of the previous studies. Haplotypes with frequencies of less than 0.03 were omitted from the analysis. Permutation test was applied 1000 times to obtain the corrected p–value. The Linkage Disequilibrium test which includes D′ and R^2^ is as shown in Figure 1.

**Table 4:** ABCB1 and ABCC2 haplotypes and treatment response to Carbamazepine mono-therapy.

Haplotypes^a^	Responders(Frequencies)	Non- responders (Frequencies)	p	OR(95% C.I.)
G C A C	8.75(0.03)	11.55(0.04)	0.43	1.43 [0.59∼3.50]
G C G C	16.23(0.05)	103.38(0.34)	1.10e−20	10.06 [5.78∼17.53]
G C G T	9.26(0.03)	19.16(0.06)	0.04	2.30 [1.03∼5.14]
G T A C	17.19(0.05)	10.19(0.03)	0.24	0.62 [0.28∼1.37]
G T G C	84.82(0.26)	74.56(0.25)	0.66	0.92 [0.64∼1.32]
G T G T	27.68(0.09)	22.13 (0.07)	0.57	0.84 [0.47∼1.51]
T C A C	10.32(0.03)	1.47(0.01)	0.01	0.15[0.03∼0.84]
T C G C	73.98(0.23)	26.72(0.09)	1.69e−6	0.32 [0.20∼0.52]
T C G T	10.30(0.03)	4.72(0.02)	0.18	0.48 [0.16∼1.44]
T T G C	51.04(0.16)	15.25(0.05)	1.27e−5	0.28 [0.16∼1.44]

Order of polymorphisms: 2677G>T, 3435C>T, 1249G>A, −24C>T

a Global χ^2^ 120.11, df = 9, p = 1.27e−21

(Haplotype were omitted if the estimated haplotype probability was less than 3%)

## Discussion

The patients were recruited irrespective of race or gender. The percentage distribution of the patients based on race was reflective of the Malaysian population in the peninsular region. They have been exposed to the same environmental factors such as diet and lifestyle. Previous findings of association studies among these SNPs which were conducted in other countries were found to be different. This was most likely due to the method in which the subjects were selected. The p value for each race in association to each polymorphism as well as to the treatment outcome was determined using Chi square statistics, to rule out genetic heterogeneity. The p values for all the above mentioned parameters were >0.05. Confounding factors such as patient compliance, drug interaction and presence of preexisting diseases were eliminated. In the end of the study, it was evident that neither race (p = 0.569) nor gender (p = 0.090) played a significant role in the outcome of treatment or had any genetic association with any of the polymorphisms. In addition to this, their renal profile and liver function test were determined to exclude the failure of therapy due to poor drug metabolism or elimination. 152 patients were responders while the other 162 were non-responders to CBZ mono-therapy. Patients were considered responders if they were free of unprovoked seizures for more than a year since the initiation of the optimum dose of CBZ as a single drug therapy. Non-responders were patients who had poor seizure control with an optimum dose CBZ mono-therapy, requiring an addition of other AEDs. Failure in drug transport has been implicated in many of the studies involving resistant to treatment in numerous diseases. The ABCB1 gene has been found to be associated with treatment resistance in many of the neurological diseases which have been studied thus far. ABCB1 is a multidrug efflux pump known to be present in all excretory organs including the epithelial lining of the blood-brain barrier. This P-gp macromolecule is responsible for drug transport [18]–[23]. ABCC2 is a member of the MRP subfamily which is involved in multi-drug resistance [24]. This protein is expressed in the canalicular (apical) part of the hepatocyte and functions in biliary transport [25].

The G2677T was the first SNP discovered from the human P-gp. The association between the G2677T SNP in relation to the treatment outcome with CBZ among Europeans [11] and Indians from India [12] were found to be non-significant. In contrary to that, a similar study done on the Japanese patients with TLE [8] was found to be significant. A study done on the Chinese population of Beijing showed a high degree of linkage disequilibrium between G2677T and two other polymorphisms of the ABCB1 gene in relation to drug resistant epilepsy [26]. However the study we conducted on the Malaysian population showed that the 2677TT genotype was significantly (p<0.001) associated with CBZ resistance in the treatment of CPS. This is similar to the study conducted on the Japanese population only recruited patients with Temporal Lobe epilepsy which is responsible for most CPS and managed to obtain significant association with the outcome of treatment.

We also found that patients with 3435TT genotype were significantly less (p = 0.007) likely to respond to CBZ mono-therapy. The 3435TT genotype was associated with decreased expression of P-gp among Caucasians [27] but increased expression in the Japanese patients [28]–[29]. Two other studies conducted on Japanese [8] and Turkish [10] patients showed evidence that the 3435TT genotype was associated with drug resistant epilepsy. A study was previously conducted by another team in Malaysia which showed no association between polymorphisms of the ABCB1 gene and drug resistant epilepsy [9]. However they had recruited patients without specifying the type of epilepsy or a specific drug used for treatment. Another important confounding factor we eliminated was known drug interaction with CBZ.

We found no correlation between the G1249A of the ABCC2 gene and the outcome of treatment of patients with CPS on CBZ monotherapy. This is similar to the findings of the study conducted on German patients [30] and Chinese patients [31].

We also found no association between the −24C>T and the outcome of treatment with CBZ monotherapy. This supports the study conducted on the Japanese patients [8] and the Korean population [32]. However the −24C>T polymorphism was found to be significantly associated with drug resistant epilepsy among German patients [30].

The haplotypes of the four SNPs showed significant association with the treatment outcome CPS with CBZ monotherapy. The haplotype combination GCGC of the 2677G>T, 3435C>T, 1249G>A and −24C>T respectively was found to be extremely significant (p = 1.10e−20) with good drug response to CBZ monotherapy while the haplotype combination of TTGC was associated with poor response. Though screening the 2677GG genotype to determine good response to CBZ monotherapy was found to be significant, based on our findings, we found that screening for SNPs which are in LD was found to be a stronger marker.

There were differences in findings observed amongst studies conducted in different countries. This shows that people of different ethnic background may influence the differences in the frequencies of these haplotypes. These differences observed may also be due to the recruitment method of patients. Different types of seizures respond differently to different AEDs. It is very important to determine patient compliance to treatment as well as to rule out drug interaction. The findings of this study may be helpful in predicting the treatment outcome of patients with CPS of certain populations before CBZ is introduced so a more effective method of treatment may be advocated.

The inference drawn from this study would presumably have a tremendous impact on cost-benefit ratio. Though this would require a separate study to be conducted it is safe to say that screening patients by determining the presence of these polymorphisms would help in deciding the best treatment option to be chosen for the patient. This would in the long term reduce the morbidity among these patients.
